# Glucagon‐like peptide‐1 (GLP‐1) signalling in the brain: From neural circuits and metabolism to therapeutics

**DOI:** 10.1111/bph.15682

**Published:** 2021-11-04

**Authors:** Anita Kabahizi, Briana Wallace, Linh Lieu, Dominic Chau, Yanbin Dong, Eun‐Sang Hwang, Kevin W. Williams

**Affiliations:** ^1^ Department of Internal Medicine, Center for Hypothalamic Research University of Texas Southwestern Medical Center at Dallas Dallas Texas USA

**Keywords:** arcuate, brain, body weight, diabetes, dorsal vagal complex, energy balance, food intake, glucose, glucose metabolism, GLP‐1, hypothalamus, NTS, obesity

## Abstract

Glucagon‐like‐peptide‐1 (GLP‐1) derived from gut enteroendocrine cells and a discrete population of neurons in the caudal medulla acts through humoral and neural pathways to regulate satiety, gastric motility and pancreatic endocrine function. These physiological attributes contribute to GLP‐1 having a potent therapeutic action in glycaemic regulation and chronic weight management. In this review, we provide an overview of the neural circuits targeted by endogenous versus exogenous GLP‐1 and related drugs. We also highlight candidate subpopulations of neurons and cellular mechanisms responsible for the acute and chronic effects of GLP‐1 and GLP‐1 receptor agonists on energy balance and glucose metabolism. Finally, we present potential future directions to translate these findings towards the development of effective therapies for treatment of metabolic disease.

**LINKED ARTICLES:**

This article is part of a themed issue on GLP1 receptor ligands (BJP 75th Anniversary). To view the other articles in this section visit http://onlinelibrary.wiley.com/doi/10.1111/bph.v179.4/issuetoc

**Abbreviations**
AGRP: agouti‐related protein
CRH/CRF: corticotropin releasing hormone/corticotropin releasing factor
GIP: gastric inhibitory polypeptide/glucose‐dependent insulinotropic polypeptide
GLP‐1: glucagon‐like‐peptide 1
KO: knockout
MC_4_ receptor: melanocortin 4 receptor
mRNA: messenger RNA
NPY: neuropeptide Y
SDA: subdiaphragmatic vagal deafferentation
Sim‐1: single‐minded 1
TRH: thyrotropin releasing hormone

## 1 GLP‐1: FROM DISCOVERY TO USE AS AN OBESITY AND DIABETES THERAPEUTIC

The discovery of the first hormone, secretin in 1902, led to the search for other gut hormones that could stimulate pancreatic secretion. However, the concept or existence of such an incretin was met by scepticism and controversy over the following century (reviewed in Holst, 2019; Müller et al., 2019; Rehfeld, 2018). Briefly, the development of radioimmunoassay methods for insulin as well as glucagon greatly advanced the detection of these hormones and allowed for in‐depth investigations on how they are regulated in normal physiology as well as disease (Scherer & Newgard, 2020). The ability to directly and reliably measure circulating insulin levels led to the description that oral glucose administration results in considerably higher insulin responses when compared with intravenous glucose, heralding in a renewed interest in incretins. The first identified incretin hormone was gastric inhibitory polypeptide (GIP), also known as glucose‐dependent insulinotropic polypeptide. Subsequent and parallel interests in the hormone glucagon suggested the existence of another incretin hormone later identified as a pro‐hormone, pro‐glucagon structure with two glucagon‐like‐peptides. This ultimately led to the discovery of glucagon‐like‐peptide 1 (GLP‐1).

**FIGURE 1:**
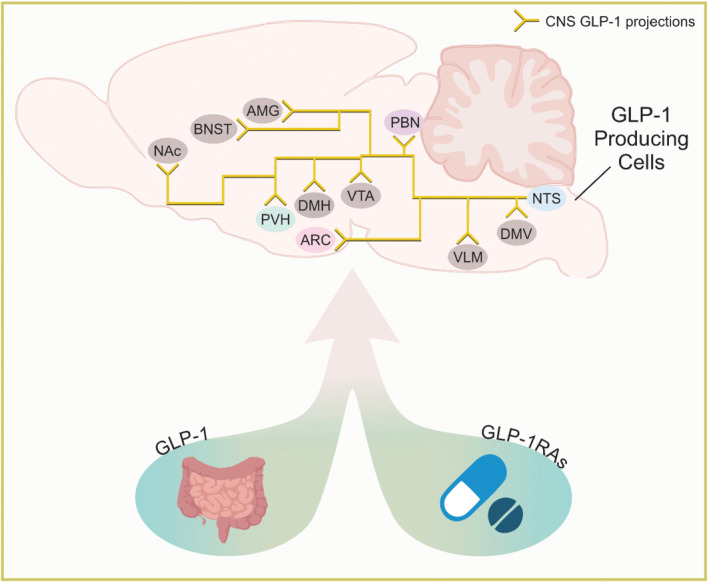
Sagittal view of a murine brain depicting select nucleus tractus solitarius (NTS|GLP‐1 projections discussed within the review. Endogenous GLP‐1 (left) and GLP‐1 receptor analogues (GLP‐1As) (right) act on a variety of brain regions. Abbreviations: AMG, amygdala; ARC, arcuate nucleus; BNST, bed nucleus of the stria terminalis; DMH, dorsomedial hypothalamus, DMV, dorsal motor nucleus of the vagus; GLP‐1, glucagon like peptide 1; GLP‐1RAs, GLP‐1 receptor agonists; NAc, nucleus accumbens; NTS, nucleus tractus solitarius; PBN, parabrachial nucleus; PVH, paraventricular hypothalamic nucleus; VLM, ventrolateral medulla and VTA, ventral tegmental area

GLP‐1 is a small peptide hormone, which is a post‐translational cleavage product of the preproglucagon encoded gene, *GCG*. GLP‐1 is produced by intestinal L‐cells and also by a discrete population of neurons in the caudal medulla (Figure 1) (Drucker, 2018; Larsen, Tang‐Christensen, Holst, & Orskov, 1997). The multiple physiological effects of GLP‐1 make it a viable candidate for diabetes mellites and obesity therapies. In particular, GLP‐1 has potent effects on blood glucose by either stimulating glucose induced insulin release or inhibiting glucagon secretion (Drucker, 2018; Holst, 2019; Müller et al., 2019), both of which limit hepatic glucose production, which has been associated with hyperglycaemia in type 2 diabetic patients. Additionally, GLP‐1 suppresses appetite and food intake (Drucker, 2018; Shah & Vella, 2014; van Bloemendaal et al., 2014). However, the beneficial effects of GLP‐1 were limited in clinical trials of type 2 diabetic patients due to the very short half‐life of GLP‐1, which is approximately 2–5 min, via degradation by the enzyme dipeptidyl peptidase 4 (DPP‐4) (Holst, 2019; Müller et al., 2019). Orally active inhibitors of DPP‐4 and long‐acting injectable/oral analogues of GLP‐1 (e.g. exendin‐4, sitagliptin, liraglutide, semaglutide, albiglutide, dulaglutide and others) were subsequently developed to enhance the efficacy of GLP‐1 (Holst, 2019; Kanoski et al., 2016; Müller et al., 2019). Physiological and pharmacological data have shown that activation of the GLP‐1 receptors promotes insulin secretion from pancreatic beta cells and also causes weight loss and thus representing a significant pharmacological target for the treatment of type 2 diabetes (Drucker, 2018; Kanoski et al., 2016). Importantly, these effects are shared across species from rodents to humans, as peripheral GLP‐1 administration to normal and diabetic human subjects induced satiety and reduced food intake in short term studies (Flint et al., 1998; Gutzwiller et al., 1999; Toft‐Nielsen et al., 1999; Verdich et al., 2001). Additionally, chronic GLP‐1 or GLP‐1 mimetic administration to human diabetic subjects was associated with improvements in glycaemic control and a modest 1.5–5.3 kg weight loss over a period of 0.5–3 years (Klonoff et al., 2008; Riddle et al., 2006). As GLP‐1 receptor agonists are effective anti‐diabetic/weight control agents and their use is rapidly expanding. It is critical to understand how GLP‐1 mediates beneficial effects on food intake/body weight and glucose homeostasis in order to develop therapies with potential for even greater efficacy and tolerability in patients. This review highlights the emerging findings that illustrate how GLP‐1 receptor signalling in the CNS reduces both food intake and body weight (with an emphasis on GLP‐1 action within the hypothalamus).

## 2 DISTRIBUTION AND REGULATION OF THE GLP‐1 SYSTEM

Although GLP‐1 deficiency maybe unlikely to contribute to impaired insulin action in type 2 diabetes (Gribble & Reimann, 2021), low GLP‐1 levels are a potential risk factor in the development of type 2 diabetes (Lastya et al., 2014). Weight gain may also lead to dysregulation of the GLP‐1 system and contribute to the maintenance of metabolic dysfunction (Ranganath et al., 1996). Moreover, type 2 (non‐insulin‐dependent) diabetic patients exhibit a reduced incretin effect (Nauck et al., 1986). Thus, better defining the factors that regulate the production and release of GLP‐1, along with the physiological conditions associated with the rise and fall of GLP‐1 levels may advance our understanding of GLP‐1 physiology in metabolic disease.

Secretion of GLP‐1 is regulated by multiple factors involved in homeostasis (Gribble & Reimann, 2021; Müller et al., 2019). Some of these factors overlap with the regulation of both the peripheral and central GLP‐1 system. For instance, in a fasted or interprandial state, GLP‐1 is secreted from enteroendocrine L‐cells at low levels (Orskov et al., 1996). Owing to the incretin actions of GLP‐1, GLP‐1 secretion is increased and circulating levels are elevated several folds in magnitude in response to a meal, which contributes to increased insulin secretion (Campbell & Drucker, 2013; Drucker & Nauck, 2006). Elevated blood levels of GLP‐1 following a meal may depend upon mechanical forces such as gastric distension (Müller et al., 2019; Rowlands et al., 2018). Nucleus tractus solitarius (NTS) GLP‐1 neurons also are activated in response to gastric distension (Vrang et al., 2003). These effects may involve oxytocin signalling on vagal afferents and contribute to the negative energy balance of central GLP‐1 (Brierley et al., 2021; Cheng et al., 2020; Gaykema et al., 2017; Holt et al., 2019; Scott et al., 2011). In addition to mechanical forces, classical satiety factors such as cholecystokinin (CCK) increase both central GLP‐1 cellular activity and peripheral GLP‐1 release (Beglinger et al., 2010; Hansen & Holst, 2002; Hisadome et al., 2011). However, the concentration of cholecystokinin necessary for peripheral GLP‐1 secretion may require non‐physiological doses (Hansen & Holst, 2002). The adipose‐derived peptide leptin also stimulates the release of GLP‐1 from rodent and human L‐cells (Anini & Brubaker, 2003). Nucleus tractus solitarius GLP‐1 neurons express leptin receptors and activation of these neurons by leptin may contribute to leptin‐induced control of food intake and body weight (Cheng et al., 2020; Elias et al., 2000; Scott et al., 2011). Similarly, 5‐hydroxytryptamine (5‐HT; serotonin) signalling is likely to influence nucleus tractus solitarius GLP‐1 neuronal activity with resulting actions on metabolism (D'Agostino et al., 2018; Holt et al., 2019). In addition to mechanical, peptide and neurotransmitter induced GLP‐1 secretion; GLP‐1 release in the periphery is also influenced by a variety of nutrients—carbohydrates, lipids, proteins and amino acids (Bodnaruc et al., 2016; Müller et al., 2019). However, less is known about nutrient‐induced changes in central GLP‐1 neuronal activity.

GLP‐1 acts through a G‐protein–coupled receptor expressed on pancreatic beta cells and neurons of the central and peripheral nervous system (Kanoski et al., 2011; Nakagawa et al., 2004; Paternoster & Falasca, 2018; Sandoval et al., 2008; Vahl et al., 2007). GLP‐1 receptors are highly abundant in circumventricular organs (CVOs) as well as nuclei involved in the regulation of energy balance (e.g. the control of food intake and energy expenditure) and glucose metabolism (e.g. glycolysis, gluconeogenesis, glycogenolysis and glycogenesis) (Knudsen & Lau, 2019; Morita & Miyata, 2012; Secher et al., 2014).

**FIGURE 2:**
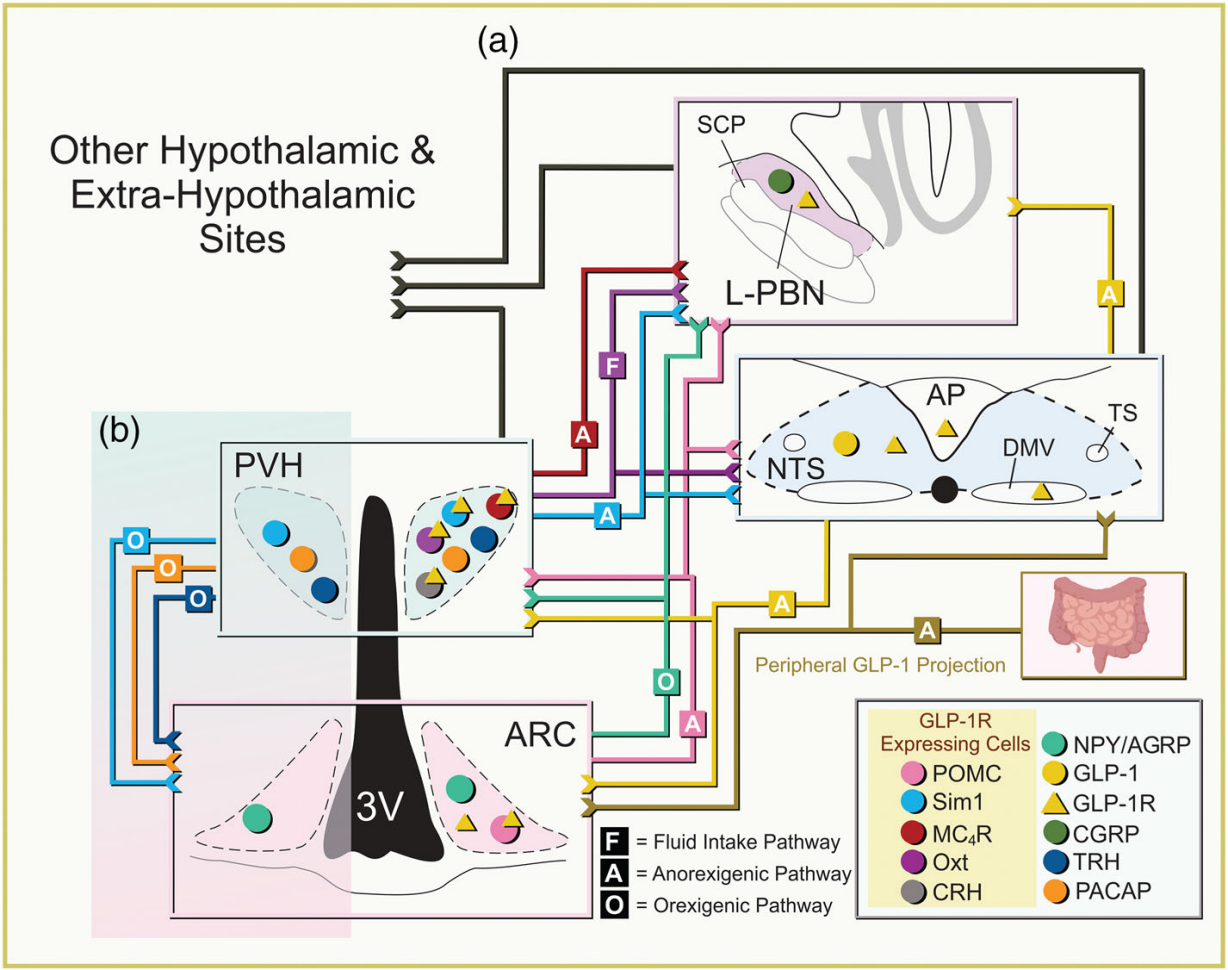
(a) Four nuclei are shown here: arcuate nucleus (ARC), lateral parabrachial nucleus neurons (L‐PBN), paraventricular hypothalamic nucleus (PVH) and nucleus tractus solitarius (NTS). Each neuronal structure has input and output projections represented by a coloured line. Each colour represents a specific cell population, that is, pro‐opiomelanocortin (POMC) is pink and projects from the ARC to the L‐PBN, nucleus tractus solitarius (NT)S and PVH. The coloured lines represent cell‐specific projections to different brain structures. Projections to a specific region are cell‐type dependent projections to that region and not to a specific cell type in that region. Projections are labelled with either F, A or O—fluid intake, anorexigenic or orexigenic pathways, respectively. Cell populations expressing GLP‐1 receptor (R)s are highlighted within a yellow box in the figure legend at the bottom right as well as with a yellow triangle on top of the GLP‐1 receptor expressing cells (coloured circle). Yellow triangles in any nuclei signify the presence of GLP‐1 receptors but not assigned to a specific cell type. Central GLP‐1 originates from the NTS (yellow) whereas peripheral GLP‐1 from the intestine (bottom right; yellow‐brown). The black arrows represent non‐cell‐type specific projections from the NTS, PVH and L‐PBN to other hypothalamic and extra‐hypothalamic sites. (b) The highlighted region focuses on the PVH single‐minded 1 (Sim1)+, pituitary adenylate‐cyclase‐activating polypeptide (PACAP)+, thyrotropin releasing hormone (TRH)+ to ARC agouti‐related protein(AGRP) orexigenic pathway (Krashes et al., 2014). Positions of cells do not represent hemispheric segregation nor exact location. Abbreviation: SCP, superior cerebellar peduncle

Within the CNS, hindbrain GLP‐1 neurons project to numerous brain regions relevant to metabolic regulation in mice (Figure 1) (Burcelin et al., 2009; Ghosal et al., 2017; Llewellyn‐Smith et al., 2011; Rinaman, 2010). Not surprisingly, these regions also express GLP‐1 receptors supporting a potential redundancy of downstream targets for effects of peripheral and/or central GLP‐1 (Figure 2) (Alhadeff et al., 2014; Brierley et al., 2020; Kanoski et al., 2016). However, it is also possible that these systems may play subtle and different roles suggesting that it is not just a matter of simple redundancy (Brierley et al., 2021).

Activation of nucleus tractus solitarius GLP‐1 neurons leads to an attenuation of metabolic rate and a reduction of food consumption in both fed and fasted states in mice (Table 1) (Cheng et al., 2020; Gaykema et al., 2017; Holt et al., 2019). Stimulation of nucleus tractus solitarius GLP‐1 neurons also suppresses glucose production without effects on glucose uptake, highlighting a potential role for the central GLP‐1 system to regulate both energy balance and glucose metabolism. (Table 1) (Gaykema et al., 2017; Shi et al., 2017). Importantly, ablation or inhibition of nucleus tractus solitarius GLP‐1 neurons increased refeeding after a fast and inhibits stress‐induced hypophagia (Holt et al., 2019). However, constitutive deficiency of pre‐proglucagon in nucleus tractus solitarius GLP‐1 neurons fails to alter energy balance (Cheng et al., 2020). Thus, there are varied reports of either continuous stimulation or loss of function/inhibition of nucleus tractus solitarius GLP‐1 neurons resulting in chronic changes in metabolism (Tables 1 and 2).

**TABLE 1:** The effect of activating or inhibiting GLP‐1 and GLP‐1 receptor expressing neurons in the brain on energy balance and glucose metabolism

Genetic marker	Acute effect	Signalling mechanism	Body weight	Lean mass	Fat mass	Food intake	EE	RER	Ambulatory activity	Blood glucose levels	ITT	Insulin sensitivity	GTT	PTT/hepatic glucose production/uptake	Taste aversion	References
**Gcg‐Cre (NTS) (JAX #000664 x BAC RP23‐242F22)**																Gaykema et al., 2017
Chow ‐ Harlan 7912	Activation	AAV2/5‐hSyn‐DIO‐hM3Dq; UNC viral core	↔	N/A	N/A	↓ Chow; ↓ fast‐refeed	↓	↔	↓	Elevated (fed state)	↔	↔	↔	Improved PTT/no change glucose uptake	↔	
DIO ‐ HCD: Research Diets D12331; DIO: Teklad TD.88137	Activation	AAV2/5‐hSyn‐DIO‐hM3Dq; UNC viral core	↓ after 5mo. DIO	↔	↓	↓ Daytime HCD sated; ↓ fast‐refeed HFD; ↓ fast‐refeed DIO	N/A	N/A	N/A	↔ (fed state)	N/A	N/A	N/A	↔	N/A	
**Gcg‐Cre (fibres in PVH) (mice from Gaykema et al.,** **2017** **)**	Activation	AAV‐EF1a‐DIO‐ChR2; Addgene#: 20298	N/A	N/A	N/A	↓	N/A	N/A	N/A	N/A	N/A	N/A	N/A	N/A	N/A	Liu et al., 2017
	Inhibition	AAV‐EF1a‐DIO‐eArch3.0; N/A	N/A	N/A	N/A	↑	N/A	N/A	N/A	N/A	N/A	N/A	N/A	N/A	N/A	
**Gcg‐cre (NTS, DMV) (mice from Gcg‐Cre cryo‐preserved embryos ID 358‐UNC) ‐** standard chow	Activation	AAV8‐hSyn‐DIO‐hM3Dq	N/A	N/A	N/A	N/A	N/A	N/A	N/A	Reduced (fasted state)	N/A	Improved	Improved	Reduced glucose production (3.5 hr post CNO)	N/A	Shi et al., 2017
**Ppg‐Cre (Gcg‐Cre ‐ mice from Gaykema et al.,** **2017** **)**																Cheng et al., 2020
Chow ‐ Purina Lab Diet, 5001	Activation	AAV‐FLEX‐hM3Dq	↓ (multi‐day tx)	N/A	N/A	↓ Overnight fast‐refeed; ↓ @ start dark cycle	N/A	N/A	N/A	N/A	N/A	N/A	N/A	N/A	N/A	
DIO ‐ HCD: Research Diets D12331; DIO: Teklad TD.88137	activation	AAV‐FLEX‐hM3Dq	N/A	N/A	N/A	↓ @ Start dark cycle	N/A	N/A	N/A	N/A	N/A	N/A	N/A	N/A	N/A	
N/A	Inhibition	AAV‐hM4Di	N/A	N/A	N/A	N/A	N/A	N/A	N/A	N/A	N/A	N/A	↔	N/A	N/A	
**Ppg‐Cre [mice from GLU‐Cre12 ‐ Parker et al.,** **2012** **] ‐** standard chow	Activation	AAV2/8‐FLEX‐hM3Dq; B. Roth	↔	N/A	N/A	↓ @ 1, 2, & 4 hr after dark onset & first 24 hr	N/A	N/A	N/A	N/A	N/A	N/A	↔	N/A	N/A	Holt et al., 2019
	Inhibition	AAV2‐FLEX‐hM4Di; B. Roth	N/A	N/A	N/A	↔ After dark onset; ↑ @ 1 hr 18 hr fast‐refeed	N/A	N/A	N/A	N/A	N/A	N/A	N/A	N/A	N/A	
**GLP‐1 receptor‐ires‐Cre (JAX #029283)**	Activation	AAV8‐hSyn‐DIO‐hM3Dq; Addgene#: 44361	N/A	N/A	N/A	↓ @ 0.5–3 hr dark onset & 16 hr fast‐refeed	↔	↔	↔	N/A	N/A	N/A	N/A	N/A	N/A	Li, Navarrete, et al, 2019
	Inhibition	AAV8‐hSyn‐DIO‐hM4Di; Addgene#: 44362	N/A	N/A	N/A	↑ @ 0.5–3 hr light cycle	N/A	N/A	N/A	N/A	N/A	N/A	N/A	N/A	N/A	
**Glp1r‐ires‐Cre/AAV‐GCaMP6s (calcium reporter)**																Li, Navarrete, et al., 2019
Chow (Teklad F6 Rodent Diet 8664)	N/A	AAV1‐CAG‐FLEX‐GCaMP6s	N/A	N/A	N/A	↑ fluorescence to food Presentation ‐ after fast refeed	N/A	N/A	N/A	N/A	N/A	N/A	N/A	N/A	N/A	
HFD (Research Diets D12492i)	N/A	AAV1‐CAG‐FLEX‐GCaMP6s	N/A	N/A	N/A	↑ Fluorescence to food presentation ‐ after fast refeed	N/A	N/A	N/A	N/A	N/A	N/A	N/A	N/A	N/A	

*Note*: ↑/↓ statistically significant response; Δ/∇ non‐significant trend; ↔ equivalent to control/ wild type.

Abbreviations: DIO, diet‐induced obese mice; DMV, dorsal motor nucleus of the vagus; EE, energy expenditure, HFD/HCD, high fat/high caloric diet; PVN, paraventricular hypothalamic nucleus; RER, respiratory exchange ratio.

**TABLE 2:** Physiological requirement of GLP‐1 receptor in the brain

Genetic marker	Body weight	Lean mass	EE	Fat mass	Food intake	GTT	ITT	References
***Glp‐1r*** ^**−/−**^								Scrocchi et al., 1996; Scrocchi & Drucker, 1998; Hansotia et al., 2007; Ayala et al., 2010
Chow (Purina 5001; Research Diets 10% kcal from fat)								
Male	↔ Up to 16 months	N/A	N/A	N/A	↔ and ↑ ( Hansotia et al., 2007 )	Impairment that subsides with age	N/A	
Female	↔ Up to 6 months; Δ at 11/16 months	N/A	N/A	N/A	↔	Impairment that persists with age	N/A	
HFD (Research Diets D12451; BioServ F3282)								
Male	Protected from weight gain (D12451 @ 9–12 weeks age); ↔ F3282 @ 3 weeks age)	↓	N/A	↔	↔ and ↑ ( Hansotia et al., 2007 )	Impaired	N/A	
Female	Protected from weight gain (F3282 @ 3 weeks age and D12451 @ 12 weeks age)	↓	N/A	↓	N/A	Impaired	N/A	
**Nestin‐Cre (JAX #3771); GLP‐1 receptor lox/lox**								Sisley et al., 2014
Chow (Harlan Teklad no. 7012)	↔	↔	N/A	↔	↔ (4 hr & 24 hr); however, separate cohorts saw ↑	Trend toward impairment	N/A	
High Fat Diet (D12331)	↔	↔	N/A	↔	↔ (4 hr, 24 hr, & 7 days)	↔	N/A	
**Vgat‐iCre, GLP‐1 receptor lox/lox**								Adams et al., 2018
Standard chow								
Male	↔	N/A	N/A	N/A	↔	N/A	N/A	
Female	↔	N/A	N/A	N/A	↔	N/A	N/A	
HFD (D12451); male only	N/A	N/A	N/A	N/A	N/A	N/A	N/A	
**Vglut‐iCre, GLP‐1 receptor lox/lox**								Adams et al., 2018
Standard chow								
Male	↔	N/A	N/A	N/A	↔	N/A	N/A	
Female	↔	N/A	N/A	N/A	↔	N/A	N/A	
HFD (D12451); male only	N/A	N/A	N/A	N/A	N/A	N/A	N/A	
**Phox2b‐Cre (JAX #016223); GLP‐1 receptor lox/lox**								Sisley et al., 2014
Chow (Harlan Teklad no. 7012)	↔	↔	N/A	↔	↔ (4 hr & 24 hr)	Trend toward impairment	N/A	
HFD (D12331)	↔	↔	N/A	↔	↔ (4 hr, 24 hr, & 7 days)	Trend toward impairment		
**Nkx2.1‐Cre (JAX #8661); GLP‐1 receptor lox/lox**								Burmeister et al., 2017
Chow (Harlan Teklad #2016)	↔	↔	↑ (48 hr)	↔	**↑**	↔	N/A	
HFD (D12492)	↓	↔	↑ (48 hr)	↓	↔	Trend toward impairment	N/A	
**POMC‐Cre (JAX #5965); GLP‐1 receptor lox/lox**								Burmeister et al., 2017
Chow (Harlan Teklad #2016)	↔	↔	↔ (48 hr)	↔	↔	↔	N/A	
HFD (D12492)	**↑**	↔	↔ (48 hr)	↔	↔	↔	N/A	
**Sim1‐Cre (JAX #6395); GLP‐1 receptor lox/lox**								Burmeister et al., 2017 Ghosal et al., 2017
Chow (Harlan Teklad #2016)	↔	↔	↓ (48 hr)	↔	↔	Trend toward improvement	N/A	
HFD (D12492)	Δ	↔	↔ (48 hr)	↔	↔	Trend toward improvement		
**PVH ‐ AAV‐Cre; GLP‐1 receptor lox/lox**								Liu et al., 2017
Standard chow	**↑**	N/A	↔	N/A	**↑**	N/A	Impaired	
HFD	N/A	N/A	N/A	N/A	N/A	N/A	N/A	
**PVH lesion**								Secher et al., 2014
Chow (no. 1324, Altromin, Brogaarden)	**↑**	N/A	N/A	N/A	N/A	N/A	N/A	
HFD	N/A	N/A	N/A	N/A	N/A	N/A	N/A	
**PpgGLP1‐NTSKO**								Cheng et al., 2020
Chow (Purina Lab Diet, 5001)	↔	↔	N/A	↔	↔	N/A	N/A	
HCD (Research Diets D12492)	N/A	N/A	N/A	N/A	N/A	N/A	N/A	
**PpgLepRbKO**								
Chow (Purina Lab Diet, 5001)	↔	↔	N/A	↔	↔	N/A	N/A	
HCD (Research Diets D12492)	N/A	N/A	N/A	N/A	N/A	N/A	N/A	

*Note*: ↑/↓ statistically significant response; Δ/∇ non‐significant trend; ↔ equivalent to control/ wild type.

Abbreviations: DIO, diet‐induced obese mice; EE, energy expenditure; HFD/HCD, high fat/high caloric diet; NTS, nucleus tractus solitarius; PVN, paraventricular hypothalamic nucleus.

The hypothalamus (a focus of this review) is a primary downstream target of peripherally administered GLP‐1 receptor agonists or hindbrain GLP‐1 neurons (Gabery et al., 2020; Müller et al., 2019). Herein, we review various brain regions, including the hypothalamus, which are required and/or sufficient to mediate the acute and chronic effects of GLP‐1 and GLP‐1 receptor agonists on energy balance and glucose metabolism. However, we acknowledge that there are differences between species (including variability in rodents) in how native peripheral/central GLP‐1 and long‐acting GLP‐1 receptor agonists alter energy balance and glucose metabolism. As our review primarily focuses on studies in mouse models, it is imperative to consider how GLP‐1 is metabolized by other species (see Section 4).

## 3 GLP‐1 SIGNALLING IN THE PERIPHERY VERSUS CNS

Classical whole animal pharmacological studies established GLP‐1 receptor activation promotes glucose tolerance and decreases food intake, thereby inducing weight loss and improving glucose homeostasis (Burcelin et al., 2009; Buse et al., 2009; Richard et al., 2014). Utilization of mouse genetic tools (see technical considerations of rat vs. mouse models) has also supported a physiological role of GLP‐1 receptors in the regulation of energy balance and glucose homeostasis. For example, global deficiency of GLP‐1 receptors consistently resulted in decreased glucose excursion in response to an oral glucose tolerance test, which was also accompanied by lowered circulating insulin levels (Table 2) (Hansotia et al., 2007; Scrocchi et al., 1996). Mice globally deficient for GLP‐1 receptors also exhibited increased blood glucose levels in response to an intraperitoneal glucose tolerance test (ipGTT) (Scrocchi & Drucker, 1998). These data support a principal action of GLP‐1 receptors to properly regulate blood glucose levels. With regard to energy balance, global deficiency of GLP‐1 receptors predictably attenuated the GLP‐1 induced hypophagia (Table 3) (Scrocchi et al., 1996). However, these mice failed to exhibit altered body weight suggesting that GLP‐1 receptors are not a key determinant of body mass under basal conditions (Scrocchi et al., 1996). Subsequent studies showed male and female mice globally deficient for GLP‐1 receptors were paradoxically protected against diet‐induced weight‐gain (Table 2) (Ayala et al., 2010; Hansotia et al., 2007; Scrocchi et al., 1996; Scrocchi & Drucker, 1998). Although not entirely clear, some of the early disparities between global knockout studies on energy balance were attributed to varying dietary components (e.g. % kcal from fat), age of diet exposure, as well as the endpoint chosen for assessment of various metabolic parameters (Ayala et al., 2010).

**TABLE 3:** Pharmacological effects of GLP‐1 receptor agonist and antagonists

Agonists	Drug	Body weight	Food intake	Fat mass	GTT	References
Genetic marker						
**Control/wild type**	GLP‐1, liraglutide, exendin‐4					Scrocchi et al., 1996; Sisley et al., 2014; Adams et al., 2018;
Standard chow		**↓**	**↓**	N/A	Improved	Burmeister et al., 2017; Ghosal et al., 2017; Secher et al., 2014;
HFD		**↓**	**↓**	**↓**	Improved	Kanoski et al., 2011; Alhadeff et al., 2012; Richard et al., 2014
***Glp1r*** ^**−/−**^	GLP‐1					Scrocchi et al., 1996
Standard chow		N/A	↔	N/A	↔	
HFD		N/A	N/A	N/A	N/A	
**Nestin‐Cre (JAX #3771); GLP‐1 receptor lox/lox**	liraglutide					Sisley et al., 2014
Chow (Harlan Teklad no. 7012)		N/A	↓ @ 4 hr; ↔ @ 24 hr	N/A	Improved	
HFD (D12331)		∇	∇ @ 7 days & 14 days	∇ (14 days)	Improved	
**Phox2b‐Cre (JAX #016223); GLP‐1 receptor lox/lox**	liraglutide					Sisley et al., 2014
Chow (Harlan Teklad no. 7012)		N/A	↓ @ 4 hr & 24 hr	N/A	Improved	
HFD (D12331)		**↓**	↓ @ 7 days & 14 days	**↓**	Improved	
**Vgat‐iCre, GLP‐1 receptor lox/lox**	liraglutide					Adams et al., 2018
Standard chow						
Male		**↓**	**↓**	N/A	N/A	
Female		**↓**	**↓**	N/A	N/A	
HFD (D12451); male only		↓ During 14 days treatment	**↓**	N/A	N/A	
**Vglut‐iCre, GLP‐1 receptor lox/lox**	liraglutide					Adams et al., 2018
Standard chow						
Male		No change @ 24 hr	↓ (Partial block)	N/A	N/A	
Female		No change @ 24 hr	↓ (Partial block)	N/A	N/A	
HFD (D12451); male only		∇ During 14 days treatment	∇	N/A	N/A	
**Nkx2.1‐Cre (JAX #8661); GLP‐1 receptor lox/lox**	exendin‐4 & liraglutide (peripheral dose)					Burmeister et al., 2017
Chow (Harlan Teklad #2016)		↓ @ 1–10 days; ∇ 11–14 days	↓	N/A	Improved	
HFD (D12492)		↓ @ 1–14 days; sig less effect from control	↓	N/A	N/A	
**Sim1‐Cre (JAX #6395); GLP‐1 receptor lox/lox**	exendin‐4 & liraglutide (peripheral dose)					Burmeister et al., 2017; Ghosal et al., 2017
Chow (Harlan Teklad #2016); Harlan (3.1 kcal/g; ∼5% fat)		N/A	↓	N/A	Improved	
HFD (D12492); Research Diets (4.54 kcal/g; ~40% fat)		N/A	↓	N/A	Improved	
**POMC‐Cre (JAX #5965); GLP‐1 receptor lox/lox**	exendin‐4 (peripheral dose)					Burmeister et al., 2017
Chow (Harlan Teklad #2016)		N/A	↓	N/A	Improved	
HFD (D12492)		N/A	↓	N/A	Improved	
**PVH lesion**	liraglutide					Secher et al., 2014
Chow (no. 1324, Altromin, Brogaarden)		↓	N/A	N/A	N/A	
HFD		N/A	N/A	N/A	N/A	
**SDA**	exendin‐4 & liraglutide					Kanoski et al., 2011; Secher et al., 2014
Standard chow		↓ @ 1–14 days	↓ (Lira 6 hr); ↓ (lira & ex‐4 24 hr)	N/A	N/A	
High fat diet		N/A	N/A	N/A	N/A	
**lPBN injection; YFP‐PPG**	exendin‐4					Richard et al., 2014
Standard chow		**↓**	**↓**	N/A	N/A	
HFD		N/A	N/A	N/A	N/A	
**VTA injection; wild type**	exendin‐4					Alhadeff et al., 2012
Chow Purina Rodent Chow, 5001		**↓** (24 hr)	↓ @ 1 hr (sucrose); ↓ @ 24 hr (chow)	N/A	N/A	
HFD (D12492)		N/A	↔ (1 and 3 hr); ↓ (6 and 24 hr)	N/A	N/A	
**NAc core injection; wild type**	exendin‐4			N/A	N/A	Alhadeff et al., 2012
Chow Purina Rodent Chow, 5001		↓	**↓** (Sucrose ); ↑ (chow)	N/A	N/A	
HFD (D12492)		N/A	↔ (1 hr); ↓ (3, 6, and 24 hr)	N/A	N/A	
**NAc shell injection; wild type**	exendin‐4					Alhadeff et al., 2012
Chow Purina Rodent Chow, 5001		↓	↔ (Sucrose); Δ @ 3 hr (chow)	N/A	N/A	
HFD (D12492)		N/A	↔ (1 and 3 hr); ↓ (6 +24 hr)	N/A	N/A	
**Control/ wild type**						Burmeister et al., 2017; Alhadeff et al., 2012; Richard et al., 2014; Sandoval et al., 2008
Standard/rodent chow	exendin‐9 & des‐His1,Glu8‐exendin‐4 (dH‐EX)	**↑**	**↑**	N/A	Impaired	
HFD	N/A	N/A	N/A	N/A	N/A	
**I.C.V. injection**						Sandoval et al., 2008
Standard/rodent chow	des‐His1,Glu8‐exendin‐4 (dH‐EX)	N/A	N/A	N/A	Impaired	
HFD	N/A	N/A	N/A	N/A	N/A	
**Nkx2.1‐Cre (JAX #8661); GLP‐1 receptor lox/lox**						Burmeister et al., 2017
Chow (Harlan Teklad #2016)	exendin‐9	N/A	N/A	N/A	Impaired	
HFD (D12492)	N/A	N/A	N/A	N/A	N/A	
**Sim1‐Cre (JAX #6395); GLP‐1 receptor lox/lox**						Burmeister et al., 2017
Chow (Harlan Teklad #2016)	exendin‐9	N/A	N/A	N/A	Trend toward impairment	
HFD (D12492)	N/A	N/A	N/A	N/A	N/A	
**lPBN injection; YFP‐PPG**						Richard et al., 2014 Alhadeff et al., 2014
Standard chow ‐ Purina Rodent Chow, 5001/standard chow	exendin‐9	**↑**	**↑**	N/A	N/A	
HFD ‐ Research Diets: 45% kcal from fat	exendin‐9	↔	**↑**	N/A	N/A	
**VTA injection; wild type**						Alhadeff et al., 2012
Chow Purina Rodent Chow, 5001	N/A	N/A	N/A	N/A	N/A	
HFD (D12492)	exendin‐9	Δ	**↑** (3 and 6 hr)	N/A	N/A	
**NAc core injection; wild type**						Alhadeff et al., 2012
Chow Purina Rodent Chow, 5001	N/A	N/A	N/A	N/A	N/A	
HFD (D12492)	exendin‐9	↔	**↑** (1 and 3 hr)	N/A	N/A	
**NAc shell injection; wild type**						Alhadeff et al., 2012
Chow Purina Rodent Chow, 5001	N/A	N/A	N/A	N/A	N/A	
HFD (D12492)	exendin‐9	↔	↔	N/A	N/A	

*Note*: ↑/↓ statistically significant response; Δ/∇ non‐significant trend; ↔ equivalent to control/ wild type.

Abbreviations: DIO, diet‐induced obese mice; EE, energy expenditure; HFD/HCD, high fat/high caloric diet.; IPBN, lateral parabrachial nucleus; NAc, nucleus accumbens; POMC, pro‐opiomelancortin; SDA, subdiaphragmatic vagal deafferentation; VTA ventromedial hypothalamus.

Specific to the brain, intracerebroventricular (i.c.v.) administration of GLP‐1 fails to alter eating in mice globally deficient for GLP‐1 receptors (Table 3) (Scrocchi et al., 1996). Inhibition of GLP‐1 receptor in the brain also impedes glucose homeostasis and food intake, whereas inactivation of GLP‐1 receptor in the gut impairs glucose‐stimulated insulin secretion, reduces glucose clearance, increases levels of glucagon and increases gastric emptying after disruption of GLP‐1 action but not food intake or body weight after 24 h (Table 3) (Knauf et al., 2008; Sandoval et al., 2008; Scrocchi et al., 1996). These reports are consistent with the demonstration that GLP‐1 receptors in the brain are capable of controlling food intake and body weight (Drucker, 2018; Gribble & Reimann, 2021; Knudsen & Lau, 2019; Müller et al., 2019; Shah & Vella, 2014). This is in addition to the hypophagic effects of endogenous GLP‐1 via peripheral GLP‐1 receptors (Ruttimann et al., 2009). As endogenous GLP‐1 is rapidly degraded once it enters circulation, hypothalamic GLP‐1 receptors are likely primarily targeted by nucleus tractus solitarius GLP‐1 neurons (Kanoski et al., 2016; Richard et al., 2015). However, recently developed long‐acting GLP‐1 analogues have also been demonstrated to target multiple nuclei within the brain, including the hypothalamus (Gabery et al., 2020; Williams et al., 2009).

It is important to note that although subdiaphragmatic vagal afferent deafferentation in rats inhibits the effects of liraglutide and exendin‐4 to suppress food intake and body weight at low doses, subdiaphragmatic vagal deafferentation fails to attenuate the food intake and body weight lowering effects of these GLP‐1 receptor agonists at high doses (Table 3) (Kanoski et al., 2011). Similarly, peripheral administration of exendin‐4 suppressed food intake and body weight equally in rats that underwent lesioning of the chemoreceptor trigger zone (CTS) for emesis ‐ the area postrema (Baraboi et al., 2010). Moreover, the hypophagic effects of the GLP‐1 agonists were blunted by administration of GLP‐1 receptor antagonists into the brain ventricular system (i.c.v. injection into the 3^rd^ ventricle; Table 3) (Kanoski et al., 2011). Together, these data suggest the potential requirement of GLP‐1 receptors in both the vagal afferents as well as the CNS for the full effects of long‐acting designer GLP‐1 receptor analogues on energy balance.

The aforementioned studies relied upon pharmacology and broad spectrum genetic tools (e.g. whole animal knockouts) to elucidate the effects of GLP‐1 on energy balance and glucose metabolism. Although these studies were greatly informative in demonstrating among other things that direct microinjection of GLP‐1 into the brain (either via administration into the ventricles or hypothalamic as well as extrahypothalamic nuclei—discussed below) may result in decreased food intake/weight gain and improved blood glucose control, it may be difficult to determine the sites of action in intact animals. Examining cellular activity directly linked or associated with physiology might also be disconnected. As a means of circumventing this dilemma, researchers used cyclization recombination‐locus of X over P1 (Cre‐loxP) technology alone or in combination with pharmacological approaches to (1) interfer with circuits, guided by neuroanatomic information coupled with the power of mouse genetics and (2) assess effects on energy and glucose homeostasis in awake, unrestrained mice.

Deficiency of GLP‐1 receptors in the CNS (both neuronal and glial cells) or in the visceral nerves failed to alter food intake of rodents when fed either a chow or a high fat diet (Table 2) (Sisley et al., 2014). Similar to global knockout studies, these data suggest that CNS and vagal GLP‐1 receptors may not be necessary for the control of food intake or body weight (Sisley et al., 2014). However, separate cohorts of mice null for GLP‐1 receptors in the CNS did reveal an increase in food intake (Sisley et al., 2014). Speculatively, these effects may be highly transient or potentially compensated for with time, as no differences in cumulative body weight or body composition were observed (Sisley et al., 2014). Knockdown of GLP‐1 receptors within the CNS also failed to alter baseline blood glucose levels (Table 2), supporting a principal action for GLP‐1 in the periphery for proper basal glycaemic control (Sisley et al., 2014). Although there was a non‐significant impairment of glucose changes in response to either an intraperitoneal glucose tolerance test or oral glucose tolerance test, lack of GLP‐1 receptors in the CNS also failed to alter glucose tolerances (Table 2) (Sisley et al., 2014). In support of these data, chronic inhibition of GLP‐1 receptors potently increased food intake, while failing to alter long‐term body weight of high fat diet fed mice (Knauf et al., 2008). However, these mice were also protected from hyperinsulinaemia and insulin resistance suggesting a potential central action of GLP‐1 receptors in regulating glucose metabolism (Knauf et al., 2008). One possible explanation for this protection may involve an apparent connection between vagal afferent GLP‐1 receptors and brown adipose tissue (BAT) (Krieger et al., 2018). However, both peripheral and central GLP‐1 receptors could be involved in the mediation of these effects. Mice deficient for GLP‐1 receptors in vagal sensory and motor neurons also exhibited increased fasting glucose levels and impaired glucose tolerance (Varin et al., 2019). These data begin to illustrate a contradiction in the assessment of GLP‐1 receptors in the CNS to regulate glycaemic control. Although not entirely clear, these data may be explained in a similar vein to the global receptor knockout (KO )reports which suggested a disparity between diet composition—with utilization of standard chow (Varin et al., 2019) or high fat diets of 58% from fat in the former study and 72% from fat in the later (Knauf et al., 2008; Sisley et al., 2014).

Opposite to loss of function, peripheral administration of liraglutide provides beneficial effects on blood glucose control in both chow‐ and high fat diet fed mice (Table 3) (He et al., 2019; Secher et al., 2014; Sisley et al., 2014). Liraglutide also suppresses food intake and body weight of high fat diet fed mice (Table 3) (Li et al., 2017; Sisley et al., 2014). Similar effects have also been observed when GLP‐1 or GLP‐1 receptor agonists are administered directly to the lateral/fourth ventricles or hypothalamus (Secher et al., 2014). Importantly, loss of GLP‐1 receptors within the CNS inhibits the effects of liraglutide on body weight, however much of the effects of liraglutide to improve blood glucose control remains intact (Table 3) (Sisley et al., 2014). Recent work also suggests glutamatergic neurons, not GABAergic neurons, may be the primary cell type responsible for the liraglutide‐induced decrease in food intake and body weight (Tables 2 and 3) (Adams et al., 2018). However, GABAergic neurons may not be completely dispensable as inhibition of hindbrain GABAergic neurons attenuates the food intake and body weight reducing effects of liraglutide in rats on a high fat diet (Fortin et al., 2020). Moreover, the pharmacological effects of GLP‐1 receptor agonists to reduce food intake are inhibited in mice deficient for GLP‐1 receptors on vagal sensory and motor neurons, an effect that was variably dependent on the class of GLP‐1 receptor mimetic utilized (Varin et al., 2019). This suggests a principal action of GLP‐1 and GLP‐1 receptor agonists to improve blood glucose control via peripheral GLP‐1 receptors, but improvements in energy balance may rely on specific neural circuits. Importantly, this may not be absolute as growing evidence supports nuclei and/or cell‐type specific effects of GLP‐1 or GLP‐1 receptor agonists (with some action within the hypothalamus) to contribute to the regulation of glucose homeostasis (discussed further below).

### 3.1 Arcuate nucleus (ARC)

Classical studies implicated the mediobasal hypothalamus in the regulation of energy balance and glucose homeostasis (Morton, 2007; Myers & Olson, 2012; Schwartz & Porte, 2005; Timper & Bruning, 2017; Williams & Elmquist, 2012). The use of pharmacology and molecular genetics led to the identification of the arcuate nucleus, which contains the orexigenic neuropeptide Y (NPY)/agouti‐related protein (AGRP) and anorexic pro‐opiomelancortin (POMC) cells. These neurons are required and sufficient for counter‐regulatory action on feeding behaviour/weight gain as well as blood glucose control.

#### 3.1.1 Arcuate GLP‐1 receptor effects on energy balance and glucose metabolism

Within the arcuate nucleus, GLP‐1 receptors are co‐expressed with pro‐opiomelanocortin neurons independent of NPY/AGRP expression (He et al., 2019; Sandoval et al., 2008; Secher et al., 2014). Injection of a GLP‐1 bolus into the third ventricle increases glucose stimulated insulin secretion, whereas inhibition of central GLP‐1 receptors impairs glucose exchanges in response to an intraperitoneal glucose tolerance test (Table 3) (Sandoval et al., 2008). Similarly, a bolus of GLP‐1 or GLP‐1 receptor agonist infusion directly into the arcuate nucleus reduces hepatic glucose production and improves glucose changes in response to a glucose challenge, supporting a link with GLP‐1 receptors in the arcuate nucleus to regulate blood glucose levels (Sandoval et al., 2008). These effects occur independent of changes in feeding behaviour on a chow diet (Sandoval et al., 2008). In agreement with these data, knockdown of GLP‐1 receptor expression in arcuate pro‐opiomelancortin neurons fails to alter basal food intake or energy expenditure of mice on a chow diet (Table 2) (Burmeister et al., 2017). Although the pharmacological effects of the GLP‐1 receptor agonist, exendin‐4, to reduce food intake was also similar between knockdown and control mice (Table 3) (Burmeister et al., 2017), antagonism of GLP‐1 receptors in the arcuate nucleus inhibits the weight reducing effects of liraglutide (Secher et al., 2014). Moreover, mice deficient in GLP‐1 receptors selectively in pro‐opiomelanocortin neurons display increased high fat diet‐induced weight gain independent of changes in food intake and energy expenditure (Table 2) (Burmeister et al., 2017). This effect may be due to distinct cell‐type and/or brain region specific roles for GLP‐1 receptor signalling as well as a potential for pro‐opiomelanocortin GLP‐1 receptors to alter handling of nutrient stores independent of changing food intake or energy expenditure (Burmeister et al., 2017). These data also highlight some inconsistencies in the interpretations of the effects of GLP‐1 in the arcuate nucleus. In particular, it appears in some instances that GLP‐1 signalling in the arcuate nucleus and/or pro‐opiomelanocortin neurons may alter energy balance/glucose metabolism, whereas at other times, this may not occur. These contradictions may depend on a variety of factors, again, previously suggested in the global knockout studies (Ayala et al., 2010). However, possibly another explanation resides with how GLP‐1 modifies the activity of the melanocortin circuit. For instance, GLP‐1 receptor agonists directly activate pro‐opiomelanocortin neurons via a transient receptor potential channel 5 (TRVP5) (He et al., 2019; Secher et al., 2014). Moreover, this activity appears to be required at least in part for the weight reducing and glucose lowering effects of liraglutide (He et al., 2019). Opposite to pro‐opiomelanocortin neurons, NPY/AGRP neurons are inhibited in response to GLP‐1 receptor agonists (He et al., 2019; Secher et al., 2014). However, NPY/AGRP neurons are inhibited indirectly via a GLP‐1 receptor dependent activation of presynaptic GABA‐ergic neurons (He et al., 2019; Secher et al., 2014). Similarly, arcuate pro‐opiomelanocortin neurons also receive an indirect enhanced excitatory tone in response to GLP‐1 receptor agonism (He et al., 2019). In many of the aforementioned genetic and pharmacological studies, the indirect regulatory activity of arcuate pro‐opiomelanocortin and/or NPY/AGRP neurons remains intact. That is, if GLP‐1 receptors are selectively ablated from pro‐opiomelanocortin neurons, then pro‐opiomelanocortin neurons may still be activated in response to GLP‐1 receptor agonists, albeit indirectly. A similar indirect regulation of NPY/AGRP neurons would also remain intact. This indirect regulatory activity may compensate for the cell‐specific GLP‐1 receptor deficiency (whereas this indirect regulation would be inhibited in whole arcuate or 3^rd^ ventricle antagonism studies) and contribute to some of the inconsistencies observed. This warrants further investigation.

### 3.2 Paraventricular hypothalamic nucleus (PVH/PVN)

Similar to the arcuate nucleus, classical lesion and pharmacological studies have implicated the paraventricular hypothalamic nucleus in regulation of energy balance and glucose metabolism (Andermann & Lowell, 2017; Gold et al., 1977; Leibowitz et al., 1981; Shor‐Posner et al., 1985; Sutton et al., 2016). It is important to note that the paraventricular hypothalamic nucleus receives afferent input from a variety of nuclei including the arcuate nucleus (Bouret et al., 2004a, 2004b; Bouret & Simerly, 2004; Geerling et al., 2010). In particular, arcuate pro‐opiomelanocortin and NPY/AGRP neurons both project to melanocortin 4 (MC_4_) receptor neurons within the paraventricular hypothalamic nucleus (Figure 2) (Krashes et al., 2013). This constitutes part of the melanocortin circuit, which is a principal contributor of monogenic forms of obesity and diabetes in multiple species from rodents to humans (Farooqi & O'Rahilly, 2005). Thus, in addition to a direct role of the paraventricular hypothalamic nucleus to regulate energy balance and glucose metabolism, neurons in the paraventricular hypothalamic nucleus function as an important relay for various circuits including melanocortin neurons, which contribute to proper metabolic regulation.

#### 3.2.1 Paraventricular hypothalamic nucleus GLP‐1 receptor effect on glycaemic control and energy balance

GLP‐1 receptors are widely distributed within the paraventricular hypothalamic nucleus (Figure 2) (Larsen, Tang‐Christensen, & Jessop, 1997). Similar to the activity of GLP‐1 cells in the periphery and the CNS (Daniels & Mietlicki‐Baase, 2019), GLP‐1 receptor expressing neurons in the paraventricular hypothalamic nucleus are activated in response to refeeding (Table 1) (Li, Navarrete, et al., 2019). The activation of paraventricular hypothalamic nucleus GLP‐1 receptor neurons occurs in response to both chow and high‐energy diets (Table 1) (Li, Navarrete, et al., 2019). Direct microinjection of GLP‐1 or GLP‐1 receptor analogues into the paraventricular hypothalamic nucleus potently suppresses food intake and body weight, largely independent of changes in glucose metabolism (Andermann & Lowell, 2017; McMahon & Wellman, 1997; McMahon & Wellman, 1998; Sutton et al., 2016). Accordingly, intra‐paraventricular hypothalamic nucleus antagonism of GLP‐1 receptors in the paraventricular hypothalamic nucleus increased food intake and body weight (Katsurada et al., 2014). Together, GLP‐1 in the paraventricular hypothalamic nucleus appears to contribute to proper energy balance. However, studies regarding the physiological importance of GLP‐1 to control food intake and body weight via action within the paraventricular hypothalamic nucleus has yielded some irregularities. For instance, while lesioning the paraventricular hypothalamic nucleus results in significant increases in body weight (Secher et al., 2014), the anorexigenic effects of the GLP‐1 receptor agonist liraglutide remained intact in paraventricular hypothalamic nucleus‐lesioned rodents (Tables 2 and 3) (Secher et al., 2014). In support of these data, loss of GLP‐1 receptors in single‐minded homolog 1 (Sim‐1) cells of the paraventricular hypothalamic nucleus failed to increase food intake (Table 2) (Burmeister et al., 2017; Ghosal et al., 2017). Moreover, mice with GLP‐1 receptor deficiency in the paraventricular hypothalamic nucleus (using Sim1‐Cre) exhibited normal anorectic responses to peripherally administered GLP‐1 receptor agonists (Table 3) (Burmeister et al., 2017; Ghosal et al., 2017). This may suggest that the paraventricular hypothalamic nucleus is physiologically dispensable for the metabolic actions of GLP‐1. Alternatively, GLP‐1 receptor‐expressing neurons in other brain regions are more involved and/or compensate for proper regulation of energy balance. However, this is in direct contradiction with other reports that identified the paraventricular hypothalamic nucleus as required in the control of food intake via CNS‐derived GLP‐1 (Liu et al., 2017). In particular, cell specific loss of GLP‐1 receptor‐expressing paraventricular hypothalamic nucleus neurons in adult mice increased body weight as well as elevated fasted blood glucose levels and impaired insulin sensitivity (Table 2) (Liu et al., 2017). Activation of GLP‐1 afferent fibres in the paraventricular hypothalamic nucleus that originated from the nucleus tractus solitarius also was sufficient to suppress food intake (Table 1) (Liu et al., 2017). Moreover, activation of GLP‐1 receptor expressing neurons in the paraventricular hypothalamic nucleus reduced dark cycle food intake and refeeding after an overnight fast (Table 1) (Li, Navarrete, et al., 2019). Accordingly, chemogenetic inhibition of GLP‐1 receptor expressing paraventricular hypothalamic nucleus neurons increased motivation to attain food and evoked hunger (Table 1) (Li, Navarrete, et al., 2019). Although GLP‐1 receptor specific effects in the paraventricular hypothalamic nucleus to induce changes in glucose metabolism may be secondary to effects on body weight, these data demonstrate a potent role for GLP‐1/GLP‐1 receptors in the paraventricular hypothalamic nucleus to maintain energy homeostasis. These findings may also exemplify a differing role for peripheral GLP‐1 versus central GLP‐1. Thus, it is possible that these data support the idea that peripheral and central GLP‐1 may act independently as well as additively to suppress food intake (Brierley et al., 2020).

#### 3.2.2 Substrate utilization

Substrate utilization is the preference between carbohydrate and fat during metabolic reactions. The inability to select between substrates may be linked to metabolic disorders. GLP‐1 has been implicated to modify substrate utilization. For example, exendin‐4 has recently been shown to alter substrate oxidation by promoting fat utilization (as measured using indirect calorimetry yielding measures of the respiratory exchange ratio, or respiratory quotient) in the paraventricular hypothalamic nucleus (Abtahi et al., 2019). Moreover, exendin‐4 attenuates the respiratory exchange ratio effects of ghrelin (a potent orexigenic peptide) injected into the paraventricular hypothalamic nucleus (Abtahi et al., 2019). Similar findings have been observed in the arcuate nucleus (Abtahi et al., 2016). Additionally, exendin‐4 pretreatment attenuates the combined effects of NPY and ghrelin co‐infusion into this same nucleus (Dalvi et al., 2012). Therefore, GLP‐1 in the paraventricular hypothalamic nucleus and the arcuate nucleus can spur a metabolic shift towards lipid utilization and contribute to peripheral substrate utilization.

#### 3.2.3 Candidate paraventricular hypothalamic nucleus neurons in GLP‐1 induced hypophagia

The paraventricular hypothalamic nucleus contains multiple cell populations that are involved in the regulation of metabolism [including single minded 1 (Sim1), oxytocin, corticotropin releasing hormone/corticotropin releasing factor (CRH/CRF), MC_4_ receptor, pituitary adenylate‐cyclase‐activating polypeptide (PACAP), thyrotropin releasing hormone (TRH) and prodynorphin neurons] (Figure 2) (Kishi et al., 2003; Lee et al., 2009; Simmons & Swanson, 2009). Some of these neurons project directly to the arcuate nucleus (either to AGRP or pro‐opiomelanocortin neurons), whereas others project to additional hypothalamic and extrahypothalamic sites (Figure 2) (Garfield et al., 2015; Krashes et al., 2014; Ryan et al., 2017; Sutton et al., 2020). Within the paraventricular hypothalamic nucleus, GLP‐1 receptors are expressed on candidate neurons including Sim1, MC_4_ receptor, oxytocin and corticotropin releasing hormone (CRH) neurons possibly independent of TRH and pituitary adenylate‐cyclase‐activating polypeptide neurons (Krashes et al., 2014; Li, Navarrete, et al., 2019). Independence of GLP‐1 receptor expression on paraventricular hypothalamic nucleus TRH and pituitary adenylate‐cyclase‐activating polypeptide neurons might be further supported by evidence that paraventricular hypothalamic nucleus Sim1+/TRH+/ pituitary adenylate‐cyclase‐activating polypeptide+ neurons project directly to arcuate AGRP neurons and form an excitatory orexigenic circuit (Figure 2) (Krashes et al., 2014). As GLP‐1 activates neurons within the paraventricular hypothalamic nucleus to suppress food intake (Liu et al., 2017), it is unlikely that GLP‐1 would activate this orexigenic circuit.

Oxytocin neurons in the paraventricular hypothalamic nucleus have classically been associated with feeding behaviour (Sabatier et al., 2013). This was recently supported by evidence that AGRP neurons (which when activated potently drive feeding behaviour) project to paraventricular hypothalamic nucleus oxytocin neurons (Figure 2) (Atasoy et al., 2012). Moreover, simultaneous activation of paraventricular hypothalamic nucleus oxytocin neurons and arcuate AGRP neurons blunts AGRP‐induced food intake (Atasoy et al., 2012). Accordingly, paraventricular hypothalamic nucleus oxytocin neurons are a potential candidate in the GLP‐1 induced suppression of food intake that is observed when GLP‐1 receptors are activated in the paraventricular hypothalamic nucleus. However, neuron ablation or bidirectional control of paraventricular hypothalamic nucleus oxytocin neuron activity has largely been limited to suppression of energy expenditure independent of eating (Sutton et al., 2014; Wu et al., 2012). Additionally, activation of paraventricular hypothalamic nucleus oxytocin neurons or downstream oxytocin neurons in the parabrachial nucleus (PBN) suppresses non‐caloric fluid intake while failing to influence eating (Figure 2) (Ryan et al., 2017). These downstream oxytocin neurons in the parabrachial nucleus in turn send projections to several forebrain regions [e.g. central nucleus of the amygdala, bed nucleus of the stria terminalis, organum vasculosum of the lamina terminalis (OVLT), anteroventral periventricular nucleus (AVPV) and median preoptic nucleus (MnPO)] and are predominately separate from calcitonin gene‐related peptide (CGRP) neurons in the parabrachial nucleus which decrease both food and fluid intake (Ryan et al., 2017) (the effects of GLP‐1 receptor signalling on feeding behaviour via actions in the parabrachial nucleus will be further discussed in section 3.4.1). Thus, although GLP‐1 recetors may influence eating in the paraventricular hypothalamic nucleus via oxytocin neurons under some physiological conditions, this may not be the primary candidate cell population for these responses.

Another possible candidate neuron population for the GLP‐1 receptor induced regulation of eating within the paraventricular hypothalamic nucleus are CRH neurons. In addition to potential access via peripheral GLP‐1 or GLP‐1 receptor agonists, nucleus tractus solitarius GLP‐1 neurons are monosynaptically connected to paraventricular hypothalamic nucleus CRH neurons (Liu et al., 2017). CRH suppresses arcuate AGRP neuronal activity possibly contributing to suppression of appetite (Kuperman et al., 2016). However, photoactivation of paraventricular hypothalamic nucleus CRH neurons fails to elicit synaptic activity in arcuate AGRP neurons (Krashes et al., 2014). Although it is possible that CRH may alter AGRP cellular activity independent of ionotropic induced changes in cellular activity, GLP‐1 effects on paraventricular hypothalamic nucleus CRH neurons may rely more heavily on modifying activity at target nuclei outside of the arcuate nucleus (Liu et al., 2017). Paraventricular hypothalamic nucleus CRH neuron activation by central GLP‐1 may also have a modulating/attenuating effect on GLP‐1's inhibitory effect on eating by stimulating the hypothalamus and pituitary adrenal axis (Lee et al., 2016).

GLP‐1 receptors are also expressed on MCR_4_ receptor+ neurons in the paraventricular hypothalamic nucleus (Li, Navarrete, et al., 2019). Paraventricular hypothalamic nucleus MCR_4_ receptor expressing neurons are prototypical neurons in the melanocortin induced regulation of satiety and body weight. In particular, paraventricular hypothalamic nucleus MCR_4_ receptor neurons are believed to receive the preponderance of melanocortin‐dependent orexigenic signalling from arcuate AGRP neurons (Garfield et al., 2015). These neurons project to multiple sites, including the lateral parabrachial nucleus (LPBN), ventrolateral periaqueductal grey matter (vlPAG), nucleus tractus solitarius and dorsal motor nucleus of the vagus (DMV) (Figure 2) (Garfield et al., 2015). Selective activation of paraventricular hypothalamic nucleus MC_4_ receptor neurons or MC receptor terminals in the lateral parabrachial nucleus potently suppresses feeding behaviour independent of illness‐associated appetite or aversion correlated with calcitonin gene‐related peptide expressing neurons in the lateral parabrachial nucleus (Garfield et al., 2015; Palmiter, 2018). Activation of MC_4_ receptor terminals in the nucleus tractus solitarius, dorsal motor nucleus of the vagus or ventrolateral periaqueductal grey matter failed to influence eating (Garfield et al., 2015). Silencing synaptic transmission from paraventricular hypothalamic nucleus GLP‐1 receptor and MC_4_ receptor neurons (not paraventricular hypothalamic nucleus oxytocin or CRH neurons) resulted in similar increases in body weight, fat mass, lean mass and food intake (Li, Navarrete, et al., 2019). Together, the activity‐dependent regulation of paraventricular hypothalamic nucleus MC_4_ receptor neurons, MC_4_ receptor projections to the lateral parabrachial nucleus, and the relationship of this circuit as well as associated nuclei with satiety and body weight make them a prime candidate for the hypophagic effects of GLP‐1.

It should be noted that GLP‐1 receptor expression within the paraventricular hypothalamic nucleus is dispersed over several cell populations outlined above (Figure 2). Considering the individual populations, GLP‐1 receptors overlap with ~40% of MC_4_ receptor+, ~30% of oxytocin+ and ~15% of CRH+ neurons in the paraventricular hypothalamic nucleus (Figure 2) (Li, Navarrete, et al., 2019). This may further support a more prominent role for GLP‐1 receptor induced hypophagia via MC_4_ receptor+ neurons over oxytocin+ or CRH+ neurons. This may also suggest a combined role for these subsets of neurons within the paraventricular hypothalamic nucleus to contribute to the metabolic effects of GLP‐1 signalling. Another important aspect is that each cell population (MC_4_ receptor+, oxytocin+ and CRH+) represents only a small fraction of GLP‐1 receptor expression within the paraventricular hypothalamic nucleus—typically each only take up 10%–15% of total GLP‐1 receptor expression in the paraventricular hypothalamic nucleus (Li, Navarrete, et al., 2019). However, deletion of GLP‐1 receptors from Sim1+ neurons resulted in a 70% reduction in GLP‐1 receptor mRNA from the paraventricular hypothalamic nucleus (Ghosal et al., 2017). This suggests the possibility of another cell population that may take the ‘lion's share’ of GLP‐1 receptor expression within the paraventricular hypothalamic nucleus or multiple other populations of neurons that have yet to be defined. One possible example of this could be the prodynorphin‐expressing neurons that lack MC_4_ receptors within the paraventricular hypothalamic nucleus and have been shown to act additively as well as independent of paraventricular hypothalamic nucleus MC_4_ receptor induced satiety (Li, Madara, et al., 2019). However, prodynorphin's actions within the context of GLP‐1 signalling are not well defined. Thus, although not entirely clear, there are multiple candidate populations of neurons within the paraventricular hypothalamic nucleus, which may mediate the effects of GLP‐1 on energy balance.

### 3.3 Additional hypothalamic sites of GLP‐1 action

In addition to the arcuate nucleus and paraventricular hypothalamic nucleus, peripheral administration of GLP‐1 or GLP‐1 receptor agonists may generate distinct patterns of cellular activity within several hypothalamic sites (Gabery et al., 2020; Parkinson et al., 2009). Direct injection of GLP‐1 receptor agonists into the lateral hypothalamic area (LHA), ventromedial hypothalamus (VMH) and dorsomedial hypothalamus (DMH) may also decrease food intake in rodents contributing to possible changes in body weight (Beiroa et al., 2014; López‐Ferreras et al., 2018). Importantly, the magnitude and/or absence of these effects may be dependent upon the specific GLP‐1 receptor agonist utilized (Beiroa et al., 2014). Moreover, the effects on body weight and not energy expenditure may be independent of GLP‐1 receptors in the ventromedial hypothalamus, as knockdown of GLP‐1 receptors in the ventromedial hypothalamus was not sufficient to block the effect of exendin‐4 to suppress food intake and improve glucose metabolism (Burmeister et al., 2017). Effects that might be explained by overlapping mechanisms.

Candidate neuron populations for the effects of GLP‐1 receptors have also been suggested within the lateral hypothalamic area and the dorsomedial hypothalamus. In particular, GLP‐1 may activate orexin‐expressing neurons independent of melanin concentrating hormone neurons (MCH) within the lateral hypothalamic area (Acuna‐Goycolea & van den Pol, 2004). Within the dorsomedial hypothalamus, GLP‐1 receptors are expressed on GABAergic neurons independent of NPY neurons (Lee et al., 2018). This selective expression of GLP‐1 receptor in the dorsomedial hypothalamus appears analogous to the GLP‐1 receptor‐dependent regulation of arcuate NPY neurons, as GLP‐1 receptor expression on GABAergic neurons negatively regulates NPY expression within the dorsomedial hypothalamus (Lee et al., 2018). These additional hypothalamic populations have suggested a role for GLP‐1/GLP‐1 receptors in the hypothalamic arousal system as well as brown adipose tissue (BAT) thermogenesis and adiposity.

### 3.4 Additional extra‐hypothalamic sites of GLP‐1 action

The role of the GLP‐1 system for energy balance extends beyond the hypothalamus with both local GLP‐1 neuronal projections within the dorsal vagal complex and more extensive projections to the mid‐brain, mesolimbic ventral tegmental area and the nucleus accumbens in the regulation of eating (Alhadeff et al., 2012, 2014; Reiner et al., 2018; Richard et al., 2014).

#### 3.4.1 The parabrachial nucleus (PBN)

The parabrachial nucleus is implicated in various aspects of energy balance including feeding behaviour and visceral satiety as well as visceral malaise, taste, temperature, pain and itch (Kanoski et al., 2016; Palmiter, 2018; Richard et al., 2014; Rinaman, 2010). Central nucleus tractus solitarius GLP‐1 expressing neurons send projections to the lateral and medial lateral parabrachial nucleus (lPBN and mPBN) (Alhadeff et al., 2014; Richard et al., 2014). Lateral ventricle injection of exendin‐4 results in the activation of lateral parabrachial nucleus neurons which can be blocked by exendin‐9 (a GLP‐1 receptor antagonist), suggesting the involvement of the central GLP‐1 system on energy balance via the parabrachial nucleus (Richard et al., 2014). This is further supported by the demonstration that pharmacological activation of GLP‐1 receptors in the lateral parabrachial nucleus neuron results in a reduction of food intake and body weight (Table 3) (Richard et al., 2014). Accordingly, blockade of GLP‐1 recetors in the lateral parabrachial nucleus neurons increases body weight and chow intake demonstrating that GLP‐1 receptors in the lateral parabrachial nucleus neurons are both sufficient and required to control food intake (Table 3) (Alhadeff et al., 2014; Richard et al., 2014). Moreover, consumption of palatable foods such as chocolate pellets and the motivation to eat palatable foods is reduced by intra‐parabrachial nucleus injection of GLP‐1 agonists (Richard et al., 2014). Caloric density and hedonic properties of food may be interacting with GLP‐1 receptor signalling within the lateral parabrachial nucleus neurons (Richard et al., 2014). Ablation of the parabrachial nucleus may also play a role in blunting the ability of the nucleus accumbens to raise dopamine levels in response to appetizing food (Richard et al., 2014).

#### 3.4.2 Mesolimbic reward system (MRS) nuclei and GLP‐1 signalling

GLP‐1 receptor signalling may also play an important role in the non‐homeostatic control of eating via activity within the ventral tegmental area (VTA) and nucleus accumbens (NAc). In particular, GLP‐1 expressing neurons in the caudal medulla project directly to the VTA and nucleus accumbens (Alhadeff et al., 2012). Administration of GLP‐1 receptor agonists to the ventromedial hypothalamus, nucleus accumbens core and shell of rats on a high‐energy diet results in the reduction of food intake (Table 3) (Alhadeff et al., 2012). This may also involve satiety promoting and food intake reducing effects by GLP‐1 via a reduction in the reward value of food by direct action in the mesolimbic reward system (MRS) (Alhadeff et al., 2012). It is also important to note that administration of exendin‐4 to the nucleus accumbens core and not the shell displayed a reduction of sucrose intake (Table 3) (Alhadeff et al., 2012). Conversely, blockade of GLP‐1 receptor in the ventromedial hypothalamus and nucleus accumbens core increased food intake (Table 3) (Alhadeff et al., 2012). This suggests that a physiological role of GLP‐1 signalling in the mesolimbic reward system to regulate energy balance. Moreover, the nucleus accumbens core may play a more significant role in carbohydrate intake and preference under food deprivation conditions (Alhadeff et al., 2012). Although these data highlight nutrient dependent effects of GLP‐1 in the nucleus accumbens, specific macronutrient selection or orosensory processing remains undefined (Alhadeff et al., 2012). Collectively, these data indicate that GLP‐1 receptor signalling in the mesolimbic reward system reduces food intake; however, the signalling cascade and downstream targets mediating this effect are not well established.

The hypothalamic, hindbrain and mesolimbic reward pathways discussed herein are reciprocally connected as well as with various other brain regions (including but not limited to the hippocampus, lateral dorsal tegmental area and the lateral septum). Many of these brain regions are also involved in the GLP‐1 receptor dependent regulation of energy balance (Reiner et al., 2018). Collectively, these data highlight a multi‐nodal neural circuit in the brain which is necessary and sufficient (and in some cases redundant or compensatory) for the full effects of GLP‐1 on energy balance and glucose metabolism.

## 4 TECHNICAL CONSIDERATIONS

As with all studies, there are several technical considerations that should be considered in context with the conclusions presented. First, there are species differences with regard to GLP‐1 dependent changes in food intake (Tornehave et al., 2008). For example, central GLP‐1 is a physiologically important signal in the control of eating and energy balance in rats (Alhadeff et al., 2017; Hayes et al., 2009). However, brain derived GLP‐1 in the mouse may be more responsible for stress‐induced hypophagia, limiting unusually large intake of food and remaining relatively irrelevant for the control of normal meals (Cheng et al., 2020; Holt et al., 2019). This suggests a context specific control of food intake in a physiological setting by central GLP‐1 receptors between rodent models. In a pharmacological context, food intake is minimally impacted by interference with endogenous GLP‐1/GLP‐1 receptor, while eating is decreased by activation of GLP‐1 neurons within the nucleus tractus solitarius (Cheng et al., 2020; Gaykema et al., 2017; Holt et al., 2019). These differences suggest that activation of central GLP‐1 neurons within the nucleus tractus solitarius and subsequent downstream GLP‐1 receptor containing circuits may provide a useful tool for controlling food intake.

Secondly, irregularities exist in the literature with regard to the hypothalamic versus extra‐hypothalamic requirement of GLP‐1 receptors in the control of food intake. As outlined in the current review, the hypothalamus is a target (not the only target) of endogenous GLP‐1 originating from hindbrain GLP‐1 neurons and for peripherally administered GLP‐1 receptor agonists in mice. However, owing to its rapid half‐life, the hypothalamus is not likely a target of endogenous peripheral GLP‐1 and probably not for peripherally administered exogenous native GLP‐1 (Fortin et al., 2020). In rats, several studies have shown that native GLP‐1 administered intraperitoneally (i.p.) requires intact vagal afferents to cause satiation, that is, to reduce meal size (Ruttimann et al., 2009). Consistent with these findings, inhibition of eating in response to i.p. administered native GLP‐1 in rats can be fully blocked by peripheral, but not by central administration of a GLP‐1 receptor antagonist (Williams et al., 2009). Together, these findings indicate that i.p. administered native GLP‐1 reduces food intake by acting on peripheral, most likely vagal afferent GLP‐1 receptors in rats. Thus, it becomes easy to see that translating results between species (even between rodents) is very challenging. The divergence of GLP‐1 neural circuitry between rats and mice are at least one likely culprit (Cork et al., 2015; Trapp & Cork, 2015). Together, this highlights a need to further delineate differences in GLP‐1/GLP‐1 receptor signalling between species in future investigations in order to better understand the physiology versus pharmacology of the GLP‐1 system.

Another distinction that should be considered is the reporting of short versus long term effects with respect to changes in feeding behaviour. In particular, many of the studies mentioned herein refer to the measurement of food intake at 24‐h intervals. It is important to note that 24‐h food intake is not feeding behaviour and examining shorter intervals may reveal important insights. For instance, vagal afferent GLP‐1 receptor knockdown by RNA interference in rats increased meal size and was compensated for by a decrease in meal frequency such that 24‐h food intake was not affected (Krieger et al., 2016). Moreover, intact abdominal vagal afferents are necessary for the full expression of the short‐term eating‐inhibitory effect of the i.p. administered GLP‐1 receptor agonist exendin‐4 (Labouesse et al., 2012). These findings suggest that an initial inhibition of eating after i.p. administration of GLP‐1 receptor agonists in laboratory animals is at least partly mediated by peripheral GLP‐1 receptors, presumably reflecting a major route/mechanism of action of endogenous peripheral GLP‐1. Due to the central effects of GLP‐1 receptor agonists this short‐term peripheral effect maybe subsequently overpowered by the central effect of the pharmacological substances. Thus, looking at shorter time intervals may reveal physiologically important differences with respect to satiation or satiety that are simply not reflected in 24‐h food intake measurements.

## 5 CONCLUSION AND FUTURE DIRECTIONS

In summary, there are multiple brain regions and neural circuits by which GLP‐1 controls food intake and body weight. GLP‐1 activity within these circuits may also contribute to proper blood glucose control. However, the pleiotropic nature of GLP‐1 might be subject to species variability as well as a dependence upon the source of GLP‐1 (i.e. whether GLP‐1 is derived from the periphery or CNS, both the periphery & CNS, or when using a specific designer long‐acting GLP‐1 receptor agonist). A primary focus of GLP‐1 action in the brain has revolved around hypothalamic sites of action (with an emphasis on the arcuate and paraventricular nuclei). However, there are also emerging and important roles for extra hypothalamic sites in the effects of GLP‐1, including hindbrain, midbrain and forebrain regions. Although these data highlight a growing understanding of GLP‐1 action in the brain, they also raise several questions for future investigation.

First, how do GLP‐1 neurons and their projections develop/form and what is required to maintain them throughout life? The brain regions and neural circuits involved in the central actions of GLP‐1 are highly plastic or malleable (Lieu et al., 2020). This plasticity may be triggered in response to a variety of nutrient, humoral and metabolic challenges (Lieu et al., 2020). The melanocortin circuit within the hypothalamus is a classic example of this plasticity (Bouret et al., 2004a; Bouret et al., 2004b; Bouret & Simerly, 2004; Lieu et al., 2020). Early studies suggested that trophic factors, such as leptin, guided the axon neurite outgrowth from arcuate NPY/AGRP and pro‐opiomelancortin neurons influencing the connections to downstream targets (Bouret et al., 2004a; Bouret et al., 2004b; Bouret & Simerly, 2004). Similar to arcuate melanocortin neurons, nucleus tractus solitarius GLP‐1 neurons express leptin receptors (Biddinger et al., 2020; Cheng et al., 2020). Leptin can acutely drive the activity of nucleus tractus solitarius GLP‐1 neurons, with the potential to influence metabolism (Scott et al., 2011). A less appreciated role for leptin in this circuit is the requirement of leptin receptors in the proper neurite outgrowth of nucleus tractus solitarius GLP‐1 neurons to target downstream neurons [putative oxytocin, CRH and MC_4_ receptor neurons (Li, Navarrete, et al., 2019)] in the paraventricular hypothalamic nucleus (Biddinger et al., 2020). This initial report suggests some specificity of this GLP‐1 neuronal targeting to the paraventricular hypothalamic nucleus, as axonal targeting of nucleus tractus solitarius GLP‐1 neurons to the arcuate nucleus was independent of leptin receptor expression on nucleus tractus solitarius GLP‐1 neurons (Biddinger et al., 2020). Surprisingly, restoration of the nucleus tractus solitarius GLP‐1 neuron projections to the paraventricular hypothalamic nucleus by expression of leptin receptors in nucleus tractus solitarius GLP‐1 neurons failed to normalize the physiological outcomes of impaired nucleus tractus solitarius GLP‐1 projections to the paraventricular hypothalamic nucleus (Biddinger et al., 2020). Similarly, loss of leptin receptors in nucleus tractus solitarius GLP‐1 neurons fails to alter long‐term energy balance (Table 2) (Cheng et al., 2020). Possibly, these data highlight the inherent redundancy in the nucleus tractus solitarius GLP‐1 central regulation of metabolism, however this warrants further investigation. The changes in axonal targeting of nucleus tractus solitarius GLP‐1 neurons to paraventricular hypothalamic nucleus neurons was also observed at the extremes of leptin receptor expression/signalling (i.e. either in leptin deficient or selective expression of leptin receptors in GLP‐1 neurons). Although these data are necessary and informative, it will be critical to better understand whether analogous effects are observed within a narrower window of changing leptin levels, as may occur during alterations of energy state or development in fed versus fasted states (Ramos‐Lobo et al., 2019). Also, increases in circulating leptin levels correlate with increased adiposity (Considine et al., 1996), and weight gain itself may result in impaired GLP‐1 signalling (Ranganath et al., 1996). Understanding the leptin induced suppression of GLP‐1 inputs to the hypothalamus during weight gain and/or in the obese state may provide an additional pathology of obesity and diabetes. Ultimately, understanding how the GLP‐1 to hypothalamic/extrahypothalamic circuits develop and are maintained may be a critical aspect in understanding the development of obesity and diabetes.

Second, there are several pharmaceuticals that have emerged for use in chronic weight management and the treatment of diabetes that act on metabolically relevant brain regions (Gautron et al., 2015; Yanovski & Yanovski, 2014). However, most of the weight loss from these medications takes place within the first 6 months of usage and rarely exceeds 5%–10% weight loss (Yanovski & Yanovski, 2014). Importantly, even moderate weight loss results in measurable improvements in blood sugar control, BP regulation and triglyceride levels (Magkos et al., 2016; Yanovski & Yanovski, 2014). However, work has continued in an attempt to achieve effects that are even more robust. Combination drug therapy was introduced as a way to surpass the weight loss barrier of a single medication (Gautron et al., 2015; Muller et al., 2018; Yanovski & Yanovski, 2014). This concept has recently been extended to the incretin system and has shown real promise. In particular, the beneficial effects of GLP‐1 have been combined in dual agonists (for both GLP‐1 and GIP receptors or GLP‐1 and glucagon receptors) or tri‐agonist (for GLP‐1, GIP and glucagon receptors) strategies (Capozzi et al., 2018; Tschöp et al., 2016). There has been a preference towards a single‐molecule multi‐agonist strategy due to a variety of competing complications when using a combination of mono‐agonists (Capozzi et al., 2018; Tschöp et al., 2016). These can include differing bioavailabilities, half‐lives, tissue specificity and pharmacokinetics (Capozzi et al., 2018). There are several clinical trials investigating the utility of these multi‐agonists strategies in humans and showing significant benefits on body weight, postprandial glucose levels, insulin sensitivity and cholesterol levels (Bastin & Andreelli, 2019; Mathiesen et al., 2019). In particular, dual agonists improve metabolic control by promoting weight loss and improving glucose tolerance (Thomas et al., 2020; Willard et al., 2020). Tri‐agonists in preclinical/clinical research have also demonstrated some superior beneficial effects compared to dual agonists, which include reduction of body weight, enhancement of glycaemic control and the reversal of nonalcoholic steatohepatitis (NASH) (Capozzi et al., 2018). Although the beneficial effects of these compounds likely involve activity at cognate receptors, better understanding any biases within these compounds for varying receptors and their effects on metabolism may advance development of future therapeutic interventions. Moreover, there is a need to understand the ability of these compounds to target receptors in the periphery versus the CNS (with an emphasis on specific brain regions) in order to maximize metabolic benefits. In addition to GIP and glucagon, GLP‐1 has also been implicated to interact with other hormones involved in metabolism. In particular, ghrelin, a potent orexigenic peptide, has also been implicated in appetitive motivation, energy metabolism, homeostasis and respiratory exchange ratio/respiratory quotient. Importantly, long‐acting GLP‐1 receptor agonists antagonize the metabolic effects of acylated ghrelin signalling within the paraventricular hypothalamic nucleus (Abtahi et al., 2019). These effects may also be apparent in leptin and 5‐HT systems (Holt et al., 2017), illustrating the neural integration of what was once thought of as separate neural systems regulating eating and metabolic‐related disorders. Together, this highlights a broad action across multiple nuclei and a need to simultaneously target multiple systems or receptor mechanisms in order to provide maximum pharmacological and therapeutic benefit for eating and metabolic‐related disorders.

Various genetic tools have been useful at identifying the glucoregulatory action of GLP‐1. However, most of these same genetic models—in the absence of pharmacological administration of GLP‐1 or GLP‐1 receptor mimetics—failed to identify a role for GLP‐1 receptors to alter food intake or body weight (Ayala et al., 2010; Ghosal et al., 2017; Scrocchi et al., 1996; Scrocchi & Drucker, 1998; Sisley et al., 2014). This might be due to several reasons: First, GLP‐1 receptor agonists are similar to other monoagonist therapies for chronic weight management, as they result in a modest 5%–15% weight loss in adults with obesity and/or diabetes (Klonoff et al., 2008; Riddle et al., 2006; Wilding et al., 2021). This modest change in body weight might be difficult to delineate in genetic models that examine changes in energy balance in only a few weeks of weight gain on either a standard chow or high‐energy diet. Second, most of the genetic models employed to investigate GLP‐1 receptors in energy balance were constitutive deletion models resulting in the ablation of GLP‐1 receptors in development. The hypothalamus (particularly the melanocortin circuit) is a classic model that is susceptible to up‐regulation of developmental compensatory pathways that mask food intake and body weight phenotypes (Wu & Palmiter, 2011; Xu et al., 2018). It is possible that an analogous compensation occurs in models with constitutive deficiency of GLP‐1 receptors, which prevents the identification of GLP‐1 receptors as required in the regulation of energy balance at basal levels. In support of this idea, ablation of GLP‐1 receptors in the adult (using AAV‐Cre injected directly into the paraventricular hypothalamic nucleus) resulted in marked obesity whereas constitutive deletion of GLP‐1 receptors in the paraventricular hypothalamic nucleus (using Sim1‐Cre) failed to alter energy balance (Table 2) (Burmeister et al., 2017; Ghosal et al., 2017; Liu et al., 2017). Perhaps, further examination of loss or gain of function of GLP‐1 receptors in the adult might be necessary for a better understanding of the requirements for GLP‐1 receptors in energy homeostasis. Another possibility is that GLP‐1 receptor signalling simply is not required for proper energy balance regulation in the basal state. Rather the GLP‐1 system and downstream GLP‐1 receptors must be activated—as occurs after a meal or in response to GLP‐1 receptor mimetics—in order to alter energy balance. However, this suggests that if GLP‐1 functions at least in part as a satiety factor then other satiety systems must compensate for the loss of GLP‐1 receptors to control food intake and body weight in times which GLP‐1 signalling would be activated (e.g. in response to a meal). The end result of these studies demonstrate that conventional genetic screens alone (in the absence of pharmacology) fail to realize the potential for improvements in food intake and body weight regulation of GLP‐1 signalling and highlight a need to pair pharmacology with genetic models.

Finally, GLP‐1 and designer GLP‐1 receptor agonists elicit changes in cellular activity within the brain that is localized to both pre‐ and post‐synaptic sites (He et al., 2019; Liu et al., 2017; Secher et al., 2014). Much of this activity has been assessed acutely and there are some discrepancies between *ex vivo* and *in vivo* measurements of this activity that warrant further investigation (Beutler et al., 2017; Su et al., 2017). However, less is also known about the effects of chronic GLP‐1 receptor agonist administration on the activity of these brain regions or neural circuits. This is an important area of investigation as clinically, patients are likely to undergo long‐term therapy with these compounds as opposed to a single administration. Better understanding the development/maintenance of GLP‐1 neurons, combinatorial therapy involving GLP‐1 designer agonists, as well as the acute versus chronic effects of GLP‐1 on the brain will undoubtedly be growing areas of study to better understand the therapeutic benefits of this system in metabolic disease.

### 5.1 Nomenclature of targets and ligands

Key protein targets and ligands in this article are hyperlinked to corresponding entries in the IUPHAR/BPS Guide to PHARMACOLOGY http://www.guidetopharmacology.org and are permanently archived in the Concise Guide to PHARMACOLOGY 2021/22 (Alexander et al., 2021).

## CONFLICT OF INTERESTS

No competing interests.

## Data Availability

The data that support the findings of this study are available from the corresponding author upon reasonable request.
